# Late orbital radiotherapy combined with intravenous methylprednisolone in the management of long-lasting active graves’ orbitopathy: a case report and literature review

**DOI:** 10.1007/s12020-024-03788-2

**Published:** 2024-03-22

**Authors:** Martina Verrienti, Irene Gagliardi, Luisa Valente, Antonio Stefanelli, Luca Borgatti, Elena Franco, Manlio Galiè, Marta Bondanelli, Maria Chiara Zatelli, Maria Rosaria Ambrosio

**Affiliations:** 1 Section of Endocrinology, Geriatrics & Internal Medicine, Department of Medical Sciences, University of Ferrara, Ferrara, Italy; 2 Unit of Cranio Maxillo Facial Surgery, Center for Craniofacial Deformities & Orbital Surgery—Reference Center for Rare Disease, St. Anna University Hospital of Ferrara, Ferrara, Italy; 3 Department of Radiation Oncology, St. Anna University Hospital of Ferrara, Ferrara, Italy; 4 Neuroradiology Unit, Department of Neuroscience and Rehabilitation, St. Anna University Hospital of Ferrara, Ferrara, Italy; 5 Operational Unit of Ophthalmology, St. Anna University Hospital of Ferrara, Ferrara, Italy

**Keywords:** Graves’ orbitopathy, Late orbital radiotherapy, VMAT-IGRT, Disease activity, Intravenous methylprednisolone

## Abstract

**Purpose:**

To present a case and review the literature on Orbital Radiotherapy (OR) combined with intravenous methylprednisolone, focusing on its late application in patients with long-lasting active Graves’ Orbitopathy (GO). Additionally, we suggest emerging perspective for future research in this context.

**Method:**

Relevant literature (randomized controlled studies, retrospective studies and reviews) was explored on PubMed from January 1973 to January 2024, searching “orbital radiotherapy” & “Graves disease”.

**Results:**

OR is a well-established second-line treatment for moderate-to-severe active GO, providing response rates comparable to glucocorticoids. Its anti-inflammatory effect makes OR particularly suitable for early active GO, and when combined with glucocorticoids, outcomes are synergistically improved. The emergence of the new Volumetric Modulated Arc Image-Guided Radiation Therapy (VMAT-IGRT) technique enables precise radiation delivery to the target, significantly reducing associated toxicity. This technological advancement enhances the feasibility of radiotherapy in benign diseases like GO. A retrospective study indicated that late OR in patients with long-lasting active GO may improve diplopia and visual acuity, decreasing disease activity. Our case report supports this conclusion.

**Conclusions:**

This report and literature review underscores the importance of considering late OR combined with intravenous methylprednisolone as a viable treatment option for GO patients with prolonged disease activity, emphasizing the crucial role of personalized therapy in managing GO. However, further investigations are warranted to validate this approach in cases of long-lasting active GO.

## Background

Graves’ Orbitopathy (GO) is an orbital autoimmune disorder, representing the most common extra-thyroidal manifestation of Graves’ Disease (~30% of patients) [1]. GO clinical presentation variably includes proptosis, eyelid retraction, palpebral edema, chemosis, red eye, retrobulbar pain, diplopia, strabismus and, rarely, visual loss [1]. The disease’s course is variable and sometimes unpredictable, demanding a multidisciplinary management. The EUropean Group on Graves’ Orbitopathy (EUGOGO) provides validated tools for the assessment of disease severity (EUGOGO classification) and activity (Clinical Activity Score, CAS) and categorizes GO into mild, moderate-to-severe, sight-threatening forms, which can be clinically active or inactive [2]. A thorough evaluation of disease severity includes a disease-specific quality of life questionnaire (GO-QoL) to guide treatment decisions as GO can significantly impact social and economic aspects, including work disability [2, 3]. Treatment choice depends on GO severity and activity in keeping with EUGOGO guideline recommendations. Intravenous (I.V.) methylprednisolone (MP) is recommended as first-line medical treatment in moderate-to-severe active GO, often combined with oral sodium (or mophetil) mycophenolate [2]. For non-responsive cases, alternative options include retreatment with high glucocorticoids doses, other immunosuppressant drugs, or orbital radiotherapy (OR). Surgical decompression and rehabilitative (extraocular muscle or eyelid) surgery are usually considered when the disease becomes inactive.

This report presents the successful treatment of a female patient with recurrent and long-lasting unilateral reactivating GO through the administration of late OR combined with I.V. methylprednisolone. We take this opportunity to briefly review the literature on OR, including its application in patients with long-lasting disease.

## Case report

A 52-year-old woman with right eye GO presented to our outpatient care in late 2018. The disease occurred one year earlier with progressive diplopia and proptosis. Initially, she was treated elsewhere with parenteral MP (cumulative dose = 4.5 g) and strabismus surgery. Thyroid assessment showed positive anti thyroid peroxydase antibodies and ultrasound consistent with chronic autoimmune thyroid disease with normal thyroid function. The patient’s history reported saphenectomy, appendectomy and smoking habit since age 20 (10 cigarettes per day; 15 pack/year).

In December 2018, she developed overt hyperthyroidism and positive thyreotropin receptor (TSH-R) antibodies (Table 1). Physical examination showed 27 mm right eye proptosis (Table 1), inferior palpebral retraction with associated lagophtalmos, redness and swelling of eyelids and chemosis, with persistent diplopia and impaired visual acuity (4/10). CAS was consistent with active disease (4/7). Orbital magnetic resonance showed increased extraocular muscles volume in the right eye and optic nerve stretching. GO-QoL questionnaire confirmed visual and psycho-social impairment (Visual Function, VF = 35.7, Appearance, AP = 37.5) (Table 1). The patient was treated with antithyroid drugs (methimazole, starting dose 15 mg/day) and then underwent total thyroidectomy in February 2019, followed by L-thyroxin replacement therapy, achieving euthyroidism. At the same time, she successfully quit smoking. The patient was then treated with parenteral glucocorticoid therapy with MP 500 mg once a week for 6 weeks, followed by 250 mg once a week for 6 weeks (cumulative dose of 4.5 g). A thorough risk assessment preceded steroid therapy, which was uneventful. In July 2019 she underwent orbital decompression surgery (medial and inferior walls with fat removal) when the disease was inactive and stable (CAS 1) for ~6 months, as previously indicated [4]. Post-operative check-up demonstrated a decrease in proptosis (25 mm in the right eye) and improved visual acuity (10/10). Nevertheless, after 4 months, disease reactivated and, as confirmed by computed tomography (CT) scan, the patient exhibited further increase in extraocular muscles diameter. Therefore, she was treated again with parenteral MP (cumulative dose 4.5 g), with successful symptoms resolution.

**Table 1:** GO assessment in our patient

	First referral (2018)	Last follow-up (2023)	Reference value
Age (years)	52	56	
Smoking Habit	Yes	No	
Laboratory findings			
TSH	<0.01 uU/ml	1.65 uU/ml	0.25–4.50
FT4	16.7 pg/ml	10.3 pg/ml	5.5–12.0
FT3	4.2 pg/ml	/	2.4–4.0
TSH-R Ab	7.9 U/L	<1.0 U/L	< 1.0
Visual findings			
Visual Acuity	4/10 (right eye), 10/10 (left eye)	10/10 (bilateral)	10/10
Tonometry	18 mmHg (bilateral)	19 mmHg (bilateral)	<18 mmHg
Funduscopy	Regular optic disc margins	Regular optic disc margins	Regular
Diplopia	Constant diplopia (also in the primary position of gaze)	Inconstant diplopia (not in the primary position of gaze)	Absent
GO Assessment tools			
CAS	4	0	< 3
Proptosis	27 mm (right eye)	22 mm (right eye)	< 19 mm
GO-QoL	VF 35.7, AP 37.5	VF 42.8, AP 43.75	100
Imaging findings			
	First referral (2018)	Last CT (2021)	
TC/RM	↑ medial rectus, inferior rectus and lateral rectus	↓ muscles area of ~40%,	
	Signs of edema	↓ orbital fat	

Patient’s GO characteristics at first referral to our center (2018) and at last follow-up (2023). The table reports patient’s age, smoke habit, laboratory, visual and imaging findings and GO assessment by CAS, exophtalmometry and GO-QoL questionnaire

**Fig. 1:**
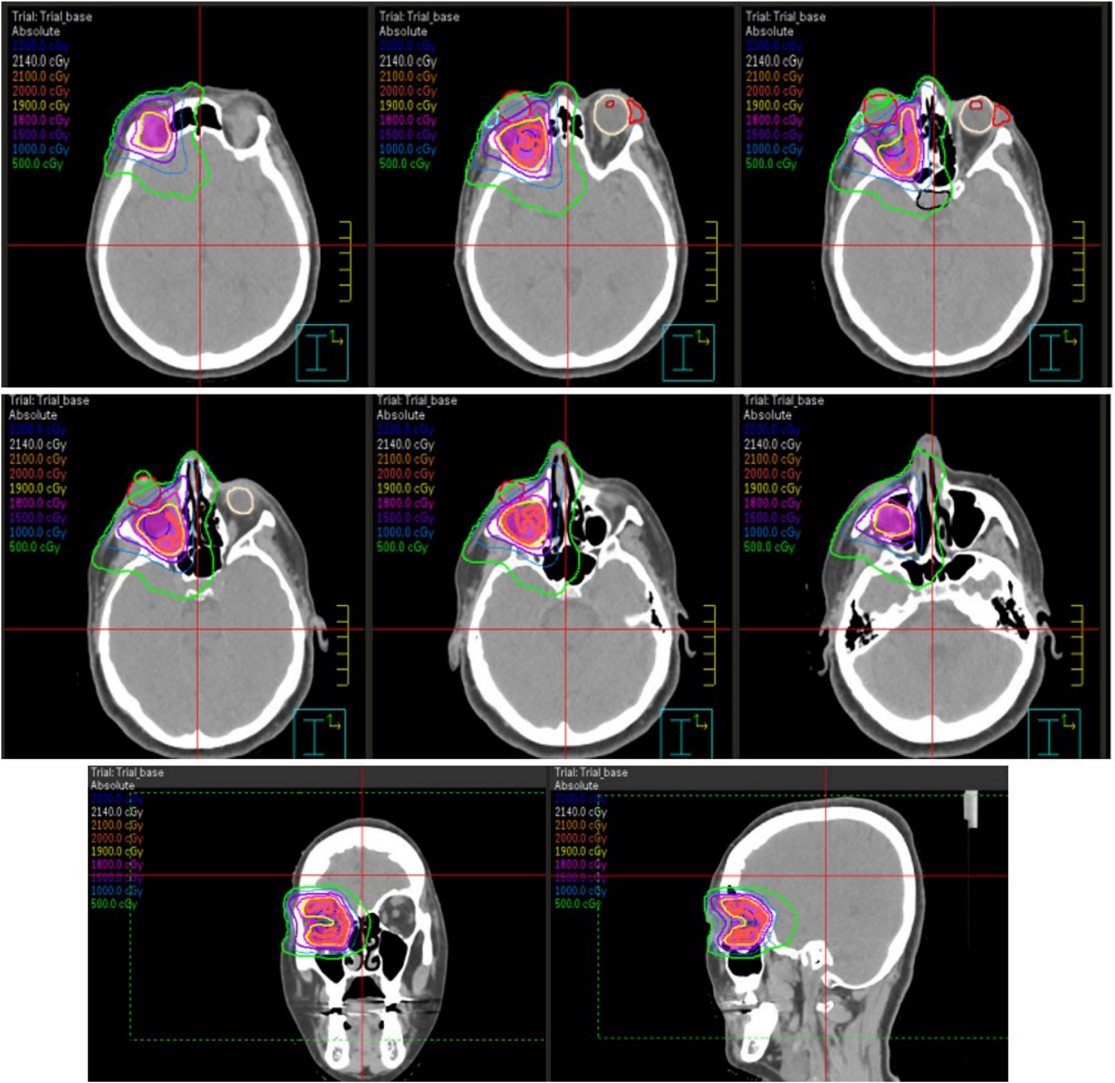
VMAT-IGRT technique. High precision X photons delivery with advanced Volumetric Modulated Arc Image-Guided Radiation Therapy

**Fig. 2:**
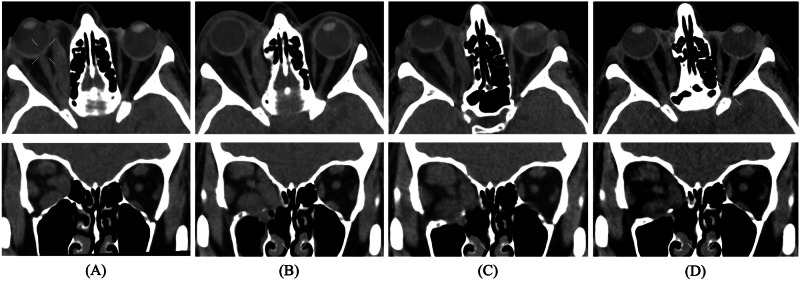
Orbital CT scans of the patient before and after treatment. Orbital CT scan (soft tissues) in axial and coronal sections before (**A**) and after (**B**) surgical decompression and before (**C**) and after (**D**) OR

**Fig. 3:**
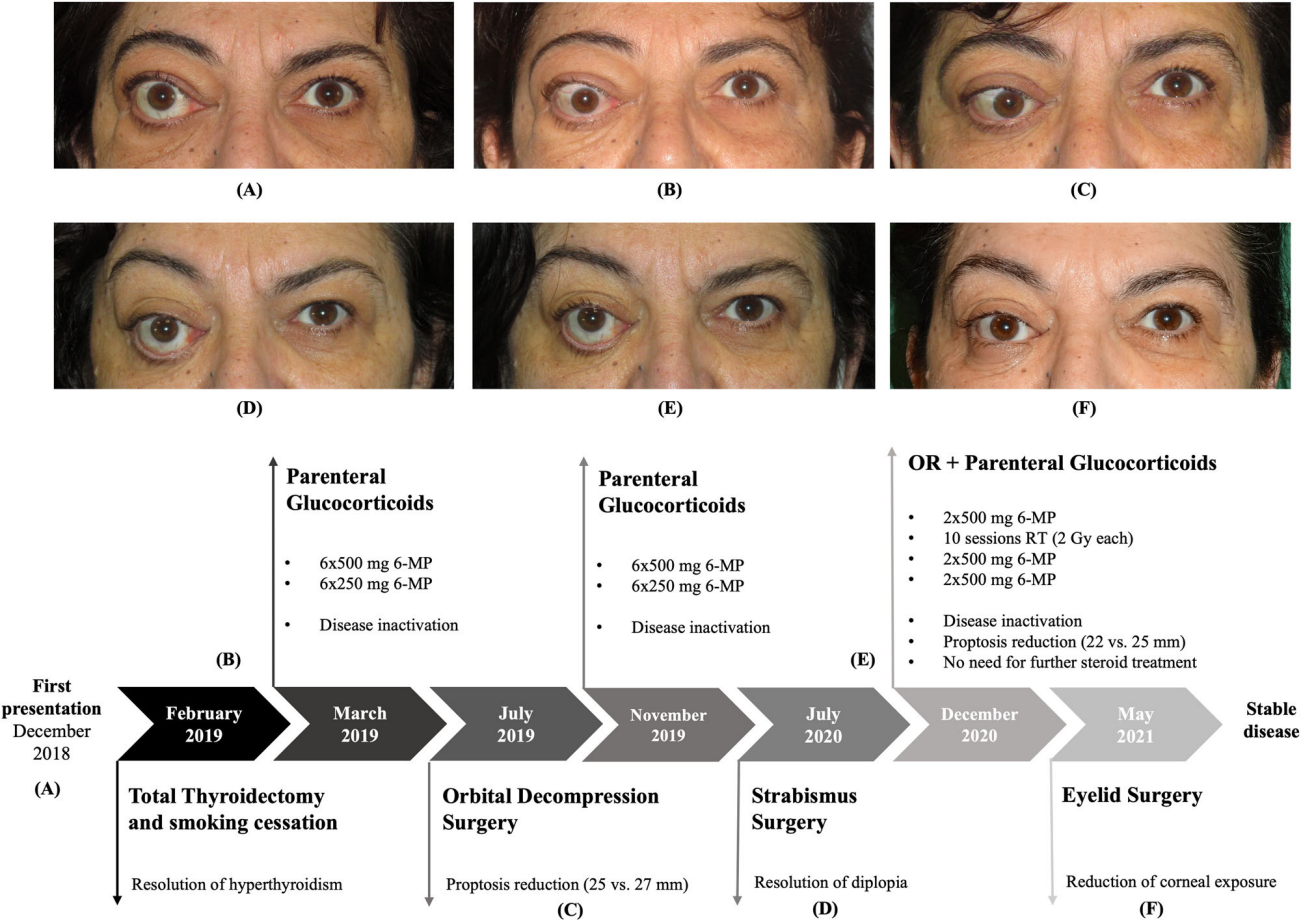
Patients’ photographs before and after treatment. **A** Patient at first presentation, (**B**) patient before steroid therapy, (**C**) patient after decompressive surgery, (**D**) patient after squint surgery, (**E**) patient before OR, (**F**) patient after OR and eyelid surgery

Subsequent strabismus surgery was required in July 2020 (CAS 1 for ~6 months). Despite abstinence from smoking and proper thyroid supplementation, signs of active GO recurred. After multidisciplinary consultation, in December 2020, 36 months after active GO onset, the patient received 10 CT-guided radiotherapy sessions (2 Gy each session) over 2 weeks, administering X photons by advanced Volumetric Modulated Arc Image-Guided Radiation Therapy (VMAT-IGRT) technique (Fig. 1). OR was associated with MP administrations: two the week before (500 mg/each) and two the week after OR (500 mg/each), followed by two further administrations of 250 mg/each in the following two weeks (cumulative dose of 2.5 g). The patient rapidly experienced resolution of GO signs and symptoms, as confirmed by a negative CAS one month after the completion of OR sessions, without side effects. At 6 months orbital CT documented reduced proptosis (22 mm), decreased muscles thickness (~40% reduction in muscle area), and decreased orbital fat tissue (Fig. 2). Physical examination at 6 and 12 months confirmed disease stabilization and lack of GO reactivation signs. Lower eyelid retractor recession with blepharoplasty followed in May 2021 (Fig. 3). By March 2023, the patient’s GO-QoL improved (VF 42.8, AP 43.75). Table 1 details her characteristics at initial referral and last follow-up in 2023.

## Literature review

### Radiotherapy techniques and mechanism of action

OR has been used for 70 years in severe and active GO, employing retrobulbar low dose radiotherapy (LD-RT), which exerts an anti-inflammatory effect [5], decreases orbital fibroblasts proliferation and glycosaminoglycans production [6]. Until few years ago, conventional RT exposed all structures within the radiation field to the same amount of radiation, posing a risk of affecting healthy surrounding tissues. Intensity-Modulated Radiation Therapy (IMRT) and, more recently, VMAT, offer improved precision and accuracy in radiation delivery by adjusting the radiation dose to the target. With VMAT (Fig. 1), the target is precisely “shaped” or “sculpted”, thus preserving healthy tissues, therefore reducing side effects. Additionally, VMAT is faster than previous approaches (5–7 min vs. 20 min per session for IMRT), improving tolerability [5]. Nowadays, toxicity is minimized, and events like cataracts and radiation retinopathy are rarely observed, with more common adverse events being dry eyes and conjunctivitis (3.9%) [7]. However, OR is not recommended for individuals under 35 years of age due to potential long-term carcinogenic risk and caution is advised in patients with severe hypertension or diabetic retinopathy due to the possible worsening of pre-existing retinal damage [8].

The conventional therapeutic approach administers 2 Gy/day for 5 days a week over 2-weeks (cumulative dose 20 Gy/orbit). However, a more fractionated delivery schedule involving 20 fractions of 1 Gy weekly over 20 weeks (20 Gy) has shown to be even more effective and better tolerated than the “short” regimen [9]. Moreover, a lower dose regimen of 10 Gy has demonstrated comparable effectiveness [9]. Over time, studies have explored very low-dose radiation regimens, showing equal efficacy compared to high-dose regimens [10]. A recent comprehensive retrospective study over a 9-year follow-up period showed that the low-dose regimen of 4.8 Gy (0.8 Gy per dose) was equally effective compared to the 20 Gy regimen in overall symptoms improvement [7]. However, patients treated with low doses required retreatment more frequently (13.1% vs. 1.7%), resulting in an increased risk of adverse events [7]. Therefore, the debate on whether to prefer a low-dose or a high-dose regimen continues. The former offers a safer profile and radiation-sparing protocol, while the latter necessitates less frequent retreatment. Ultimately, choice is left to the clinician’s discretion with a patient-tailored approach, as we did in our case.

### Efficacy of orbital radiotherapy

Several clinical studies have demonstrated OR usefulness in GO [6, 11–13]. Several studies demonstrated that OR and glucocorticoids are similarly efficacious in treating GO [14], providing similar response rates (65–70% vs. 60–80%) [12, 15] as well as satisfactory symptom control [6].

Moreover, studies suggested that combining systemic glucocorticoids with OR leads to more successful outcomes than either treatment alone [14, 16–19]. This combination demonstrates a synergistic effect and helps control transient edema that may occur during OR. However, there are no standardized guidelines regarding the administration scheme or preferred route (oral vs. intravenous) for glucocorticoids [2] (Table 2). OR has shown early efficacy on inflammatory soft tissue changes, visual acuity and eye muscle motility, although it has limited impact on proptosis and longstanding ocular motility restriction [6, 11, 20]. Nevertheless, doubts about OR efficacy have been raised [21].

**Table 2:** Glucocorticoid regimens in association with OR in the literature [11, 12, 15, 17, 18, 19, 21]

First author, Year	Study design	N° of patients	RT fractionation schedule	GC schedule
Bartalena et al., [16]	Controlled clinical trial	36	20 Gy in 2 weeks (2 Gy/session)	Oral MP 70–80 mg/day for 3 weeks, then tapered for 5-6 months
Marcocci et al., [18]	Prospective study	13	20 Gy in 2 weeks (2 Gy/session)	Oral Prednisone 100 mg/day for 7 days, then tapered for 5-6 months
Sisti et al., [12]	Retrospective study	96	10 Gy in 2 weeks (1 Gy/session)	I.V. MP ~ 6 to ~8 g (15 mg/kg × 4 times + 7.5 mg/kg × 8 times)
Kim et al., [22]	Retrospective study	68	20 Gy in 2 weeks (2 Gy/session)	I.V. MP 4.5 g (500 mg/day × 3 days + 500 mg/week × 6 weeks) before OR followed by oral prednisolone 1 mg/daily tapered for 2–3 month
Overhaus et al., [19]	Retrospective study	72	12–20 Gy in 2–4 weeks (1–2 Gy/session)	• I.V. MP 4.5 g (6 × 500 mg + 6 × 250 mg) • I.V. MP 3 g (6x500mg) • I.V. MP 2.25 g (3 × 500 mg + 3 × 250 mg) • I.V. MP 1.125 g (3 × 250 mg + 3 × 125 mg)
Nicosia et al. [13]	Retrospective study	40	20 Gy in 2 weeks (2 Gy/session)	I.V. MP 500 mg weekly

A significant aspect of OR is its potential as a steroid-sparing therapy. Kim et al. [22] and Hahn et al. [23], in studies with 9 and 12 months follow-up respectively, suggested that OR is associated with a lower recurrence rate and longer disease-free intervals, reducing the need for further steroid or immunosuppressive therapy. Additionally, Matthiesen et al. retrospective study of 211 non-responders to glucocorticoids (median follow up of 11 months) showed disease stabilization after OR without recurrence in 96.7% of patients and glucocorticoid discontinuation in 97.8% of patients [24].

Considering these findings, the EUGOGO guidelines recommend OR as a second-line treatment for moderate-to-severe active GO, in combination with glucocorticoids, particularly for patients with diplopia and/or restricted extraocular motility [2]. The 2022 American Thyroid Association and the European Thyroid Association Consensus statement recommends addressing to OR primarily patients with progressive diplopia [8].

### What do we know about orbital radiotherapy in long-lasting GO?

In long-standing and stable GO, radiotherapy often yields partial responses compared to patients with shorter duration diseases. In fact, once inflammation transitions to fibrosis, radiotherapy’s effectiveness diminishes. Studies report an inverse relationship between GO duration and OR success [24–26]. Despite this, OR swiftly resolved GO symptoms and activity in our case, though performed ~3 years post-onset. Indeed, some GO patients defy the traditional disease course experiencing prolonged activity or disease reactivation, especially when potential risk factors are not removed [25]. For these cases labeled as “long-lasting active GO”, OR stands as a suitable anti-inflammatory treatment, regardless of disease duration [27]. A recent retrospective study by Choi and Lee explored late OR efficacy in prolonged disease [27]. They included early-active (<24 months) and late-active (>24 months) patients undergoing OR due to steroids unresponsiveness or non-eligibility, revealing significant improvement in CAS, diplopia and visual acuity in both groups. Extraocular movement limitation and proptosis did not significantly improve in the late-active group [27]. The study suggests that disease duration does not necessarily hinder OR efficacy, especially in patients with high CAS or visual disturbance, though its retrospective nature limits solid conclusions. In addition, this study presents further limitations, including the relatively small number of patients, the short follow-up and the possible patients’ selection bias.

## Discussion

This report details the case of a woman with moderate-to-severe, long-lasting, recurrent and disfiguring unilateral GO who finally achieved disease control after late OR combined with I.V. MP. Although multidisciplinary approach is usually the winning strategy, GO management can be challenging, as seen in this case. Our patient experienced multiple disease flare-ups requiring many clinical assessments, surgeries and steroid treatments over a 3-year span. Despite these interventions, multiple disease reactivations prompted us to employ a combination of OR and intravenous glucocorticoids for managing her severe GO. Since GO was inactive for at least 6 months before orbital surgery, we cannot rule out the hypothesis that the latter may have had a role in disease reactivation, as previously hypothesized [28].

Consistent with the findings of Choi and Lee, this approach rapidly alleviated symptoms and effectively inactivated the disease. Furthermore, OR combined with I.V. MP significantly improved proptosis. However, further investigations are warranted to substantiate these outcomes in GO patients with prolonged reactivating disease activity. In our case, the most important aim was to achieve definitive disease stabilization, allowing for final eyelid surgery, extended follow-up intervals and steroid treatment discontinuation. These results align with existing literature [22–24]. Based on our experience, OR-associated administration of parenteral glucocorticoids is preferable over the oral route due to its superior efficacy and tolerability [2]. However, comprehensive studies are required to establish an optimal therapeutic regimen.

In summary, our case report highlights the importance of OR combined with I.V. MP as a viable treatment option for long-lasting reactivating GO, also in cases where it is applied late in the disease course. However, our study also underscores the unsolved issues, particularly the need for a standardized steroid protocol and for additional data on this subject.

## Abbreviations

GO: Graves’ Orbitopathy
EUGOGO: EUropean Group on Graves’ Orbitopathy
CAS: Clinical Activity Score
QoL: Quality of life
I.V.: Intravenous
MP: Methylprednisolone
OR: Orbital Radiotherapy
TSH-R: Thyreotropin Receptor
VF: Visual Functioning
AP: Appearance
CT: Computed Tomography
VMAT-IGRT: Volumetric Modulated Arc Image-Guided Radiation Therapy
LD-RT: Low-dose Radiotherapy
IMRT: Image Modulated Radiant Therapy
